# Antiplatelet dilemma: Clopidogrel or aspirin for long-term cardiovascular protection after dual antiplatelet therapy following PCI

**DOI:** 10.1097/MD.0000000000047773

**Published:** 2026-02-28

**Authors:** Mostafa Hossam El Din Moawad, Mahmoud Elsayed, Ibrahim Serag, Reham M. Wagih, Abdelrahman Elgharabawi, Yousr Ahmed, Abdelrahman Elkholy, Ahmed Abdelraouf, Areej M. Alsolami, Ahmed Alattar, Ibraheem M. Alkhawaldeh, Hamza A. Abdul-Hafez, Ahmed Farid Gadelmawla, Mohamed Abouzid, Mohamed O. Mohamed, Osama Bisht, Mohammed Elkholy

**Affiliations:** a Alexandria Main University Hospital, Alexandria, Egypt; b Faculty of Medicine, Suez Canal University, Ismailia, Egypt; c Stroke and Neurovascular Regulation Laboratory, Charlestown, MA; d Faculty of Medicine, Mansoura University, Mansoura, Egypt; e Department of Total Parenteral Nutrition, Alexandria Main University Hospital, Alexandria, Egypt; f Faculty of Biology, Medicine and Health, Manchester University, Manchester, The United Kingdom; g Department of Pulmonology and Critical Care, Johns Hopkins University, Baltimore, MD; h Faculty of Medicine, Alexandria University, Alexandria, Egypt; i Faculty of Medicine, Al-Azhar University, Cairo, Egypt; j Department of Biology, College of Science, Jouf University, Saudia Arabia; k King Abdullah Medical City, Makkah, Saudi Arabia; l Faculty of Medicine, Mutah University, Al-Karak, Jordan; m Department of Medicine, An-Najah National University, Nablus, West Bank, Palestine; n Faculty of Medicine, Menoufia University, Menoufia, Egypt; o Medical Research Group of Egypt (MRGE), Negida Academy, Arlington, MA; p Department of Physical Pharmacy and Pharmacokinetics, Faculty of Pharmacy, Poznan University of Medical Sciences, Poznan, Poland; q Institute of Health Informatics, University College London, London, UK; r Evangelisches Herzzentrum Coswig, Teilen Speichern, Coswig (Anhalt), Germany; s Department of Radiology, The Laboratory for Minimally Invasive Tumor Therapies, Beth Israel Deaconess Medical Center/Harvard Medical School, Boston, MA.

**Keywords:** aspirin, bleeding, clopidogrel, dual antiplatelet therapy, major adverse cardiovascular events, percutaneous coronary intervention, stroke

## Abstract

**Background:**

The optimal choice of antiplatelet monotherapy after dual antiplatelet therapy (DAPT) among patients undergoing PCI remains debatable. While aspirin has long been the default choice, clopidogrel has emerged as a potential alternative due to its lower bleeding risk and possible superior ischemic protection. This meta-analysis sought to investigate the recent findings comparing the use of aspirin against clopidogrel after different durations of DAPT in patients who underwent PCI.

**Methods:**

We searched for randomized controlled trials and cohort studies comparing long-term aspirin and clopidogrel monotherapy after DAPT post-PCI. The primary outcome was major adverse cardiovascular events (MACE). Secondary outcomes included major and minor bleeding, gastrointestinal (GI) bleeding, stroke, myocardial infarction (MI), target vessel revascularization, and stent thrombosis.

**Results:**

Six studies comprising 14,992 patients were included. Clopidogrel monotherapy was associated with a significantly lower risk of MACE than aspirin (risk ratio [RR]: 1.24; 95% CI: 1.09–1.42; *P* = .001). Aspirin monotherapy resulted in a higher risk of minor bleeding (RR: 1.57; 95% CI: 1.06–2.34; *P* = .03) and GI bleeding (RR: 1.19; 95% CI: 1.04–1.37; *P* = .01). However, aspirin monotherapy was associated with a higher risk of stroke, including both ischemic (RR: 1.56; 95% CI: 1.03–2.38; *P* = .04) and hemorrhagic stroke (RR: 2.06; 95% CI: 1.06–3.98; *P* = .03).

**Conclusion:**

Clopidogrel monotherapy appears to be a superior alternative to aspirin for long-term secondary prevention after PCI, offering a lower risk of MACE and stroke without increasing major bleeding or mortality. However, individual factors such as genetic variability, GI bleeding risk, and cost should guide therapy selection.

## 1. Introduction

Percutaneous coronary intervention (PCI) is the most utilized revascularisation strategy for patients with acute coronary syndrome (ACS) and chronic coronary syndrome. Patients undergoing PCI with drug-eluting stents (DES) commit to dual antiplatelet therapy (DAPT), comprising aspirin and a P2Y12 inhibitor (P2Y12i), for 6 to 12 months to mitigate the risk of early stent thrombosis and recurrent ischemic events.^[1–3]^ Following this interval, continuous administration of single antiplatelet treatment is recommended to prevent atherosclerotic cardiovascular events.

Previous studies and a meta-analyses have shown that P2Y12i monotherapy, mostly utilizing clopidogrel, decreased cardiovascular events relative to aspirin monotherapy without an increase in bleeding risk.^[4–8]^ There is a concern that genetic variations in cytochrome P450 2C19 enzyme (CYP2C19) may lead to significant individual variability in medication efficacy, with a considerable proportion of patients, especially those of East Asian descent, potentially exhibiting a poor response to clopidogrel.^[9–11]^ Furthermore, clopidogrel is more costly than aspirin, and cost-effectiveness may remain a concern for prolonged use, necessitating additional data to substantiate a significant ischemia advantage with P2Y12i monotherapy.^[12,13]^

The present systematic review and meta-analysis investigates the latest evidence comparing the utility and safety of long-term maintenance aspirin and clopidogrel after DAPT among patients undergoing PCI.

## 2. Methods

This meta-analysis was conducted following the Preferred Reporting Items for Systematic Reviews and Meta-Analyses (PRISMA) guidelines^[14]^ and the Cochrane Handbook for Systematic Reviews of Interventions.^[15]^ Study selection, data extraction, and quality assessment were performed independently by 2 authors, with discrepancies resolved through discussion or consultation with a 3rd author.

### 2.1. Search strategy

A comprehensive search was performed across 3 databases, PubMed, Scopus, and Web of Science, to identify all relevant studies for inclusion in this systematic review and meta-analysis. The search spanned from the inception of each database up to January 2025. We utilized Medical Subject Heading (MeSH) terms related to the keywords “Aspirin” AND “Clopidogrel” AND “Percutaneous coronary intervention” AND “Monotherapy” OR “Alone” OR “Comparison” OR “Compared” OR “Versus” OR “VS.” The search strategy was tailored to each database: for PubMed, the search was conducted using titles and abstracts; for Scopus, the search included keywords, titles, and abstracts; and for Web of Science, the search was limited to abstracts. The retrieved articles were imported into EndNote for duplicate removal before proceeding to the screening phase.

### 2.2. Eligibility criteria and screening process

After removing duplicates, the remaining articles were uploaded to Rayyan for screening. The screening process followed the Population, Intervention, Comparison, Outcome (PICO framework) and was conducted in 2 stages: title and abstract screening, followed by full-text screening. We included studies in our review if they satisfied the following criteria:

1. Population (P): patients who completed DAPT after PCI.
2. Intervention (I): clopidogrel monotherapy.
3. Comparison (C): aspirin monotherapy.
4. Outcomes (O):
  (i) primary outcome: major adverse cardiovascular events (MACE).
  (ii) Secondary outcomes: major and minor bleeding according to Thrombolysis In Myocardial Infarction (TIMI) criteria, Bleeding Academic Research Consortium (BARC) bleeding scale 2, 3, and 5, BARC bleeding 3 or 5, GI bleeding, all-cause mortality, cardiovascular mortality, stroke including ischemic and hemorrhagic stroke, myocardial infarction (MI), target vessel revascularization (TVR), and stent thrombosis.
5. Study design: we included randomized controlled trials (RCTs) and cohort studies.

### 2.3. Data extraction

Data extraction was performed using Microsoft Excel (Microsoft Corporation, Redmond). The extracted data included baseline characteristics and a summary of the studies (e.g., study design, country, sample size, age, gender, duration of DAPT, dose of the drugs, ACS and non-ACS, follow-up and definition of MACE) and outcome measures including event and total of MACE, major and minor bleeding according to TIMI criteria, BARC bleeding scale 2, 3, and 5, BARC bleeding 3 or 5, GI bleeding, all-cause mortality, cardiovascular mortality, stroke including ischemic and hemorrhagic stroke, MI, TVR, and stent thrombosis.

### 2.4. Quality and risk of bias assessment

For observational cohort studies, the Newcastle–Ottawa Scale (NOS) was used. The NOS assigns a rating ranging from 0 to 9, with studies rated 1 to 3 stars: low quality, 4 to 6 stars: moderate quality, and 7 to 9 stars: high quality.^[16]^ The Cochrane Risk of Bias Tool (RoB-2) was employed for RCTs. This tool evaluates 5 domains, and the overall risk of bias is categorized as low risk, some concerns, or high risk.^[17]^

### 2.5. Data analysis and synthesis

Statistical analyses were performed using Review Manager Software (The Cochrane Collaboration, London, United Kingdom, England). Pooled risk ratio (RR) was used for dichotomous variables to calculate the risk of different outcomes using their event and total. Regarding the heterogeneity, the chi-square test evaluated statistical heterogeneity among studies. Then, the chi-square statistic was used to calculate *I*^2^. A Chi-square with a *P*-value <.1 was considered significant heterogeneity. Also, the *I*^2^ value of more than or equal to 50% indicated high heterogeneity. The random-effects model was applied for outcomes with significant heterogeneity, while the fixed-effects model was used for outcomes with non-significant heterogeneity. Sensitivity analysis was performed using the leave-one-out method, where 1 study was removed at a time to assess its impact on heterogeneity. Subgroup analysis according to study design was conducted.

## 3. Results

### 3.1. Searching and screening

**Figure 1.**
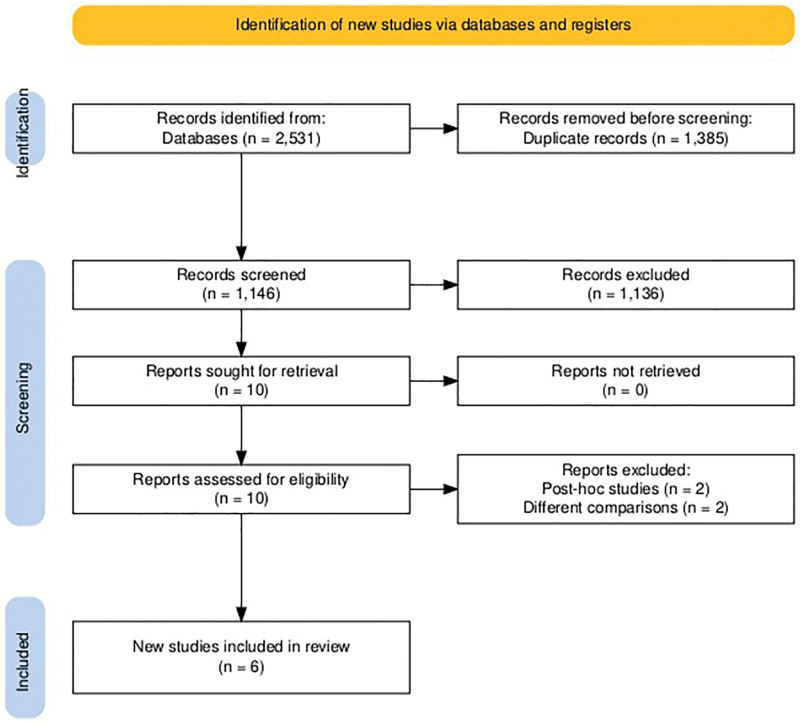
PRISMA flow diagram of searching and screening processes. PRISMA = Preferred Reporting Items for Systematic Reviews and Meta-Analyses.

After title and abstract screening, 2531 articles were retrieved from the included database. Among them, 1146 articles were involved in the title and abstract screening after removing 1385 duplicates. Ten articles were eligible for full-text screening, including 6 in the current systematic review and meta-analysis.^[5,18–22]^ (Fig. 1).

### 3.2. Summary of the included studies

Table 1 summarizes the key features of the included studies, including the duration of DAPT, doses of aspirin and clopidogrel, the proportion of patients with ACS versus non-ACS, follow-up periods, and primary outcomes.

**Table 1:** Summary of the included studies.

Study	Duration of DAPT		Dose		ACS, n (%)		No ACS, n (%)		Follow-up	MACE
	Aspirin	Clopidogrel	Aspirin	Clopidogrel	Aspirin	Clopidogrel	Aspirin	Clopidogrel		
Zhuang^[19]^	12 mo		100 mg/day	75 mg/day	NR	NR	NR	NR	2 years	Cardiac death, MI, or TVR.
Park^[21]^	12 mo		NR	NR	1017 (41.1)	324 (42)	1455 (58.9)	447 (58.0)	3 years	Cardiac death, MI, or stroke.
Sim^[22]^	12 mo		100 mg/day	75 mg/day	NR	NR	NR	NR	1 year	A composite of death from any cause, MI, repeat PCI, stent thrombosis, or ischemic stroke.
Lan^[20]^	12 mo		100 mg/day	75 mg/day	507 (87.1)	355 (84.1)	75 (12.9)	67 (15.9)	2 years	Composite of cardiac death, ischemic stroke, MI, and BARC bleeding type 3 or greater.
Koo^[5]^	6–18 mo		100 mg/day	75 mg/day	1957 (71.7)	1965 (72.5)	771 (28.3)	746 (27.5)	2 years	A composite of all-cause death, non-fatal MI, stroke, readmission due to ACS, and BARC bleeding type 3 or greater.
Watanabe^[18]^	12 mo	1 mo	81–200 mg/day	75 mg/day	573 (38.6)	557 (37.9)	913 (61.4)	914 (62.1)	5 years	A composite of cardiovascular death, MI, definite stent thrombosis, or any stroke.

ACS = acute coronary syndrome, BARC = Bleeding Academic Research Consortium, DAPT = dual antiplatelet therapy, MI = myocardial infarction, NR = not reported, PCI = percutaneous coronary intervention, TVR = target vessel revascularization.

The duration of DAPT varied across studies, with most studies employing a 12-month DAPT period, except for Koo^[5]^, which had a variable DAPT duration of 6 to 18 months, and Watanabe^[18]^ with only 1 month in the clopidogrel group. Aspirin doses ranged from 81 to 200 mg/day, while clopidogrel was consistently administered at 75 mg/day. The proportion of ACS patients varied significantly across the studies. For example, Lan^[20]^ reported a high proportion of ACS patients (87.1% in the aspirin group and 84.1% in the clopidogrel group), while Watanabe^[18]^ reported a lower proportion (38.6% in the aspirin group and 37.9% in the clopidogrel group). Conversely, the proportion of non-ACS patients ranged from 12.9% (Lan^[20]^) to 62.1% (Watanabe^[18]^). The primary outcome of these studies was predominantly MACE, though the specific definitions varied. Follow-up periods ranged from 1 year to 5 years. The study by Watanabe^[18]^ had the most prolonged follow-up period (Table 1).

### 3.3. Baseline characteristics and summary of the included studies

Table 2 provides a detailed overview of the baseline characteristics of the 6 included studies, which consisted of 4 cohort studies and 2 RCTs. These studies were conducted in various countries, including China, Korea, and Japan, and enrolled 14,992 patients. The sample sizes for the aspirin and clopidogrel groups were relatively balanced across the studies, with the smallest study (Zhuang^[19]^) including 283 patients in the aspirin group and 248 in the clopidogrel group, and the largest study (Koo^[5]^) including 2728 and 2710 patients in the aspirin and clopidogrel groups, respectively. The mean age of participants ranged from 60.7 years (Sim^[22]^) to 70.1 years (Zhuang^[19]^). Most participants were male, with percentages ranging from 73.3% to 80.2% in the aspirin groups and 73.9% to 78.8% in the clopidogrel groups (Table 2).

**Table 2:** Baseline characteristics of the included studies.

Study	Design	Country	Sample size		Age (yr), mean (SD)		Male, n (%)	
			Aspirin	Clopidogrel	Aspirin	Clopidogrel	Aspirin	Clopidogrel
Zhuang^[19]^	Cohort	China	283	248	68.3 (7.8)	70.1 (6.2)	NR	NR
Park^[21]^	Cohort	Korea	2472	771	62 (3.7)	64 (4.1)	1811 (73.3)	570 (73.9)
Sim^[22]^	Cohort	Korea	1285	534	60.7 (11.7)	62.2 (11.7)	1030 (80.2)	395 (74.0)
Lan^[20]^	Cohort	China	582	422	62.62 (12.44)	66.38 (11.67)	453 (77.8)	321 (76.1)
Koo^[5]^	RCT	South Korea	2728	2710	63.4 (10.7)	63.5 (10.7)	2039 (74.7)	2015 (74.4)
Watanabe^[18]^	RCT	Japan	1486	1471	69 (10.4)	68.1 (10.9)	1138 (76.6)	1159 (78.8)

NR = not reported, RCT = randomized controlled trial, SD = standard deviation.

### 3.4. Quality and risk of bias assessment

According to NOS, the 4 cohort studies were deemed high quality (Table S1, Supplemental Digital Content, https://links.lww.com/MD/R419). The 2 RCTs assessed by Rob-2 were of low risk of bias (Figure S1, Supplemental Digital Content, https://links.lww.com/MD/R419).

### 3.5. Meta-analysis

**Figure 2.**
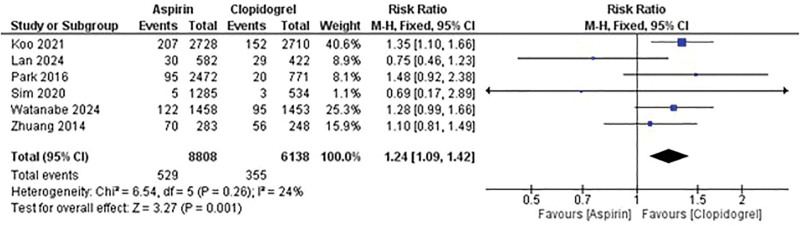
Comparison between aspirin and clopidogrel monotherapy in the risk of MACE. MACE = major adverse cardiovascular events.

The comparative analysis between aspirin and clopidogrel monotherapy revealed significant differences in the risk of MACE. Aspirin monotherapy was associated with a higher risk of MACE compared with clopidogrel, with RR of 1.24 (95% CI: 1.09–1.42; *P* = .001) (Fig. 2).

**Figure 3.**
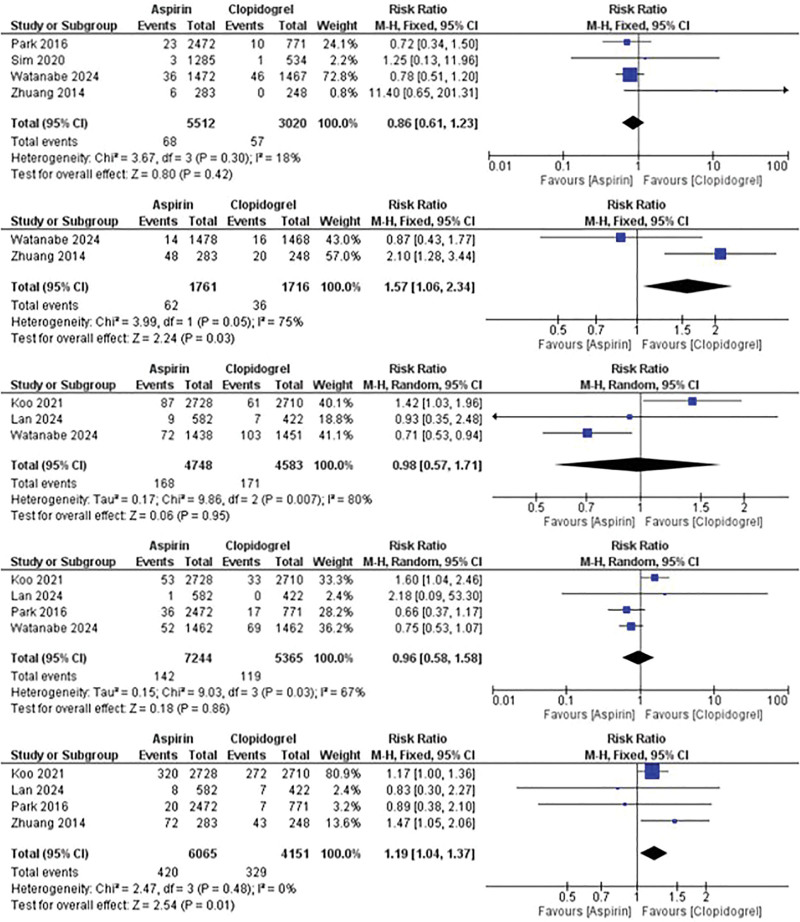
Comparison between aspirin and clopidogrel monotherapy in the risk of major bleeding (TIMI), minor bleeding (TIMI), BARC bleeding 2, 3 or 5, BARC bleeding 3 or 5, and GI bleeding (from top to bottom respectively). BARC = Bleeding Academic Research Consortium, GI = gastrointestinal, TIMI = thrombolysis in myocardial infarction.

In terms of bleeding risks, no significant difference was observed between aspirin and clopidogrel in the risk of major bleeding (TIMI) with RR of 0.86 (95% CI: 0.61–1.23; *P* = .42); however, aspirin showed a higher risk of minor bleeding (TIMI) compared with clopidogrel (RR: 1.57; 95% CI: 1.06–2.34; *P* = .03). On the other hand, no significant difference was observed between the 2 drugs regarding the risk of BARC bleeding 2, 3, or 5 (RR: 0.98; 95% CI: 0.57–1.71; *P* = .95) with significant heterogeneity as *I*^2^ = 80%, *P* = .007 and risk of BARC bleeding 3 or 5 (RR: 0.96; 95% CI: 0.58–1.58; *P* = .86) and significant heterogeneity with *I*^2^ = 67%, *P* = .03. However, GI bleeding was more significantly observed in aspirin than clopidogrel, showing RR of 1.19 (95%CI: 1.04–1.37; *P* = .01) (Fig. 3).

**Figure 4.**
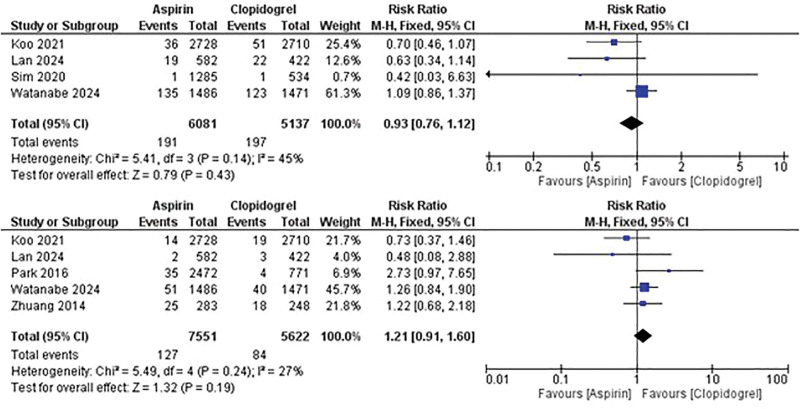
Comparison between aspirin and clopidogrel monotherapy in the risk of mortality (top) and cardiovascular mortality (bottom).

Figure 4 highlights the mortality outcomes, with no significant difference observed in all-cause mortality between the 2 therapies (RR: 0.93; 95% CI: 0.76–1.12; *P* = .43) in addition to the risk of cardiovascular mortality (RR: 1.21; 95% CI: 0.91–1.6; *P* = .19).

**Figure 5.**
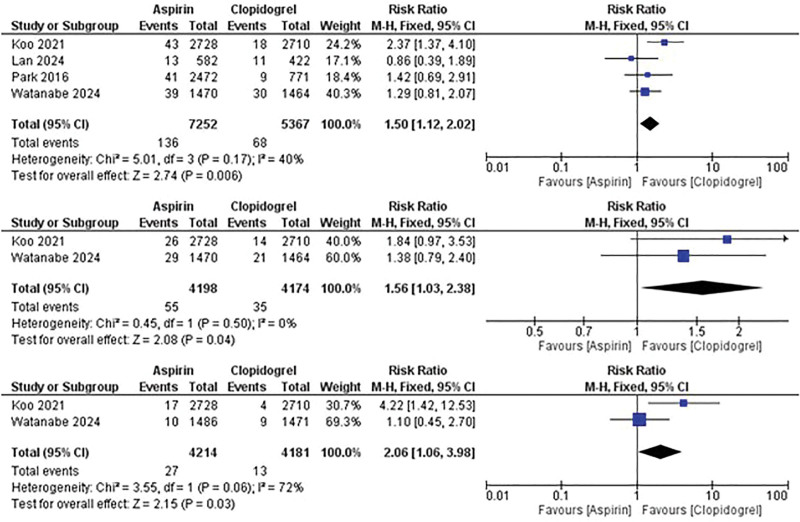
Comparison between aspirin and clopidogrel monotherapy in the risk of stroke, ischemic stroke, and hemorrhagic stroke (from top to bottom respectively).

Regarding stroke outcomes, aspirin monotherapy was associated with an increased risk of stroke (RR: 1.5; 95% CI: 1.12–2.02; *P* = .006) whether ischemic (RR: 1.56; 95% CI: 1.03–2.38; *P* = .04) or hemorrhagic (RR: 2.06; 95% CI: 1.06–3.98; *P* = .03) compared with clopidogrel (Fig. 5).

No significant difference was observed between aspirin and clopidogrel regarding the risk of MI (RR: 1.25; 95% CI: 0.98–1.57; *P* = .07), TVR (RR: 1.06; 95% CI: 0.77–1.47; *P* = .72), or stent thrombosis (RR: 1.36; 95% CI: 0.58–3.21; *P* = .48) (Figures S2–S4, Supplemental Digital Content, https://links.lww.com/MD/R419).

Sensitivity analysis using the leave-one-out method was done for the heterogeneous outcomes and showed that Watanabe was the primary source of heterogeneity in the BARC outcomes due to the difference in duration of DAPT compared with other studies (Figures S5 and S6, Supplemental Digital Content, https://links.lww.com/MD/R419).

Subgroup analysis showed that clopidogrel was associated with lower risk of MACE compared with aspirin in the RCT subgroup (RR: 1.32; 95% CI: 1.13–1.55; *P* = .0005), while they were comparable in the cohort studies subgroup (*P* = .49). Similar findings were observed in the risk of MI outcome as clopidogrel was associated with lower risk of MI compared with aspirin (RR: 1.58; 95% CI: 1.33–2.21; *P* = .008) according to RCTs but no difference was obtained in the cohort studies (*P* = .85), however, no significant difference was obtained between both groups in the risk of cardiac mortality with no effect of subgroups (Figures S7–S9, Supplemental Digital Content, https://links.lww.com/MD/R419, respectively).

## 4. Discussion

This systematic review and meta-analysis compared aspirin and clopidogrel monotherapy following DAPT in post-PCI patients. The findings suggest that clopidogrel monotherapy is associated with a significantly lower risk of MACE and stroke compared with aspirin, without an increase in major bleeding or all-cause mortality. While aspirin was linked to a higher risk of minor and GI bleeding, clopidogrel demonstrated a more favorable antithrombotic effect. No significant differences were observed in MI, TVR, or stent thrombosis risks. These results suggest that clopidogrel may be the preferred long-term monotherapy option in post-PCI patients. However, individual factors such as genetic variability, cost, and bleeding risks must be considered in clinical decision-making.

It is essential to recognize that antiplatelet monotherapy following the discontinuation of DAPT is required to be a lifelong regimen.^[1,23–25]^ Aspirin is currently the conventional treatment following the discontinuation of DAPT, and its effectiveness in avoiding cardiovascular events has been well-documented in patients with coronary artery disease (CAD) or cerebrovascular illness.^[26]^ Aspirin is a low-cost medication regarded as cost-effective for lifelong secondary prevention of cardiovascular events.^[12,13]^ Nonetheless, concerns persist regarding GI mucosal adverse effects associated with low-dose aspirin, resulting in the common coadministration with a proton pump inhibitor (PPI), as seen by the prevalence of concomitant PPI use in up to 79% of patients.^[18]^ Short-term aspirin administration results in gastric ulcers, anemia, and GI bleeding. Inhibition of prostaglandin synthesis has been suggested as a possible mechanism for GI bleeding. Aspirin diminishes platelet aggregation and vasoconstriction via decreasing the formation of thromboxane A2. Aspirin’s effect on platelet cyclooxygenase is irreversible, extending the lifespan of platelets to 7 to 10 days. Repeated administration of aspirin results in a cumulative impact on platelet function, alongside its irritation of the stomach mucosa. The concurrent ulcerogenic and antithrombotic properties predispose individuals to the bleeding tendencies frequently seen with aspirin. Randomized controlled trials regarding aspirin-associated GI bleeding indicate that the risk of peptic ulcer disease is 1.3, whereas the risk of upper GI symptoms is 1.7. Fatal hemorrhages are uncommon, and toxicity correlates with dosage.^[27,28]^

Clopidogrel has been rigorously studied as a substitute for aspirin, offering enhanced antiplatelet efficacy with less GI mucosal adverse effects. Clopidogrel has historically been widely utilized as an antiplatelet monotherapy for patients with peripheral artery disease or stroke, while aspirin has consistently served as the primary antiplatelet monotherapy for those with CAD. This distinction arises from the considerable variability in clopidogrel’s efficacy among the 3 subgroups of peripheral artery disease, stroke, and MI, as demonstrated in the clopidogrel versus aspirin in patients at risk of ischemic events (CAPRIE trial).^[4]^ The Harmonizing Optimal Strategy for Treatment of Coronary Artery Diseases-Extended Antiplatelet Monotherapy trial recently established the superiority of clopidogrel over aspirin in mitigating cardiovascular events in patients who sustained DAPT without clinical incidents for 6 to 18 months post-PCI with DES.^[5,6]^ Despite the substantial evidence favoring the ischemic advantages of clopidogrel over aspirin, current guidelines advocate for aspirin as the standard antiplatelet monotherapy beyond 1-year post-PCI. Furthermore, in the recent European Society of Cardiology 2023 ACS guidelines, the endorsement for clopidogrel was classified as Class IIb.^[1,23–25]^ Consequently, additional evidence is required to endorse clopidogrel monotherapy as a lifelong treatment for patients with CAD.

Determining the correct duration of DAPT is crucial for balancing the risks of ischemic and hemorrhagic consequences following DES placement. The duration of DAPT varied among the included trials, with 12 months being the predominant duration, except in 2 studies where it was 6 to 18 months in 1^[5]^ and only 1 month prior to clopidogrel in the other.^[18]^ Meta-analyses indicate that short-term DAPT lasting <12 months is linked to comparable ischemic event rates and reduced bleeding rates following DES insertion compared to a 12-month duration.^[29,30]^ The efficacy and safety of prolonged DAPT exceeding 12 months have recently been assessed in the DAPT trial^[31]^ and the PEGASUS-TIMI54 trial, which investigates the prevention of cardiovascular events in patients with a history of MI using a reduced dosage regimen of ticagrelor compared to placebo alongside aspirin.^[32]^ The 2 trials exhibited a thrombotic advantage of prolonged DAPT. Nonetheless, the mitigation of MI risk did not result in a mortality advantage, and a heightened incidence of bleeding events was noted in the 2 studies. Due to the potential for bleeding associated with prolonged DAPT beyond 12 months, it is crucial to ascertain the appropriate treatment for patients necessitating a single antiplatelet agent, either aspirin or clopidogrel. The CAPRIE trial has been the sole extensive investigation comparing aspirin monotherapy to clopidogrel monotherapy in patients with atherosclerotic vascular disease.^[4]^ The CAPRIE trial demonstrated that clopidogrel exhibited a marginally significant benefit over aspirin in preventing stroke, MI, and vascular disease among 19,185 patients with a recent stroke, MI, or peripheral artery disease (annual event rate 5.32% vs 5.83%; *P* = .043). Moreover, clopidogrel was linked to a reduced occurrence of upper GI pain and GI bleeding compared to aspirin. Despite clopidogrel’s superior efficacy and favorable safety profile, aspirin remains recommended due to its marginally statistically significant inferiority and cost-effectiveness; approximately 200 patients must use clopidogrel instead of aspirin for 1 year to prevent a single vascular event. Clopidogrel shown to have suboptimal cost-effectiveness for the secondary prevention of coronary heart disease in the United States.^[12]^ However, cost-effectiveness studies, primarily conducted in European nations, indicated that clopidogrel is deemed appropriate for secondary prevention in individuals with atherosclerotic vascular disorders.^[33]^ Furthermore, the new availability of clopidogrel in a generic version may alleviate its high cost in in developing economies.

The results of this meta-analysis have important implications for clinical practice. While aspirin has traditionally been the standard of care for long-term secondary prevention after PCI, the findings suggest that clopidogrel may be a more effective and safer alternative, particularly in patients at high risk of ischemic events or stroke. The higher risk of MACE and stroke with aspirin, coupled with its increased risk of minor and GI bleeding, suggests that clopidogrel may be a preferred option for many patients. However, the choice of antiplatelet therapy should be individualized based on patient characteristics, including the risk of bleeding, history of stroke, and cost considerations. Clopidogrel is generally more expensive than aspirin, which may be a concern in resource-limited settings. Additionally, genetic variations in CYP2C19, which affect clopidogrel metabolism, may influence its efficacy in specific populations. Carriers of CYP2C19 loss-of-function (LOS) alleles exhibit reduced clopidogrel metabolism, leading to lower active metabolite levels and impaired platelet inhibition. This variability is particularly prevalent in East Asian populations, with up to 30% of individuals classified as poor metabolizers. As a result, the efficacy of clopidogrel may be compromised in these patients, potentially diminishing its benefits over aspirin. Genotyping or platelet function testing may offer personalized guidance in selecting the optimal antiplatelet regimen. In such cases, alternative P2Y12i, such as ticagrelor or prasugrel, may be considered, although these agents are associated with a higher risk of bleeding.^[34]^

For translation into clinical practice, the selection of long-term antiplatelet monotherapy must be aligned with patient-specific characteristics, health system resources, and physician prescribing patterns. Evidence suggests that clopidogrel monotherapy may be particularly beneficial in patients at high ischemic risk with low bleeding susceptibility, as well as those with cerebrovascular comorbidities.^[35]^ However, in settings where CYP2C19 genotyping is not routinely implemented, clinicians may adopt a risk stratification approach based on clinical risk factors and platelet reactivity when available.^[36,37]^ Practical strategies for implementation include: genotype-guided antiplatelet selection, now recommended by multiple pharmacogenomic consortia for patients with ACSs undergoing PCI,^[38,39]^ use of PPIs when aspirin is preferred, especially in patients with prior peptic ulcer history or advanced age, to reduce GI bleeding without attenuating cardiovascular benefit,^[40]^ shared decision-making models that incorporate patient preferences, treatment cost, long-term adherence capacity, and access to follow-up monitoring,^[41]^ and integration of antiplatelet selection into discharge pathways, multidisciplinary heart team meetings, and electronic clinical decision support systems.^[42]^

In addition to the overall treatment effect observed in this meta-analysis, it is critical to recognize that the comparative benefit of clopidogrel versus aspirin is not uniform across all patient groups. Several clinical variables including age, diabetes mellitus, chronic kidney disease, prior ischemic stroke, prior GI bleeding, and baseline platelet reactivity may significantly modify antiplatelet responsiveness and therefore influence the risk-benefit calculation of monotherapy choice.^[43,44]^ For example, patients with a prior history of ischemic stroke or peripheral vascular disease may derive enhanced ischemic protection from clopidogrel, whereas patients with a history of upper GI bleeding may be more vulnerable to aspirin-related mucosal injury.^[45]^ Furthermore, CYP2C19 genotype status plays a central role in clopidogrel metabolism and antiplatelet efficacy; poor metabolizers have higher platelet reactivity and increased ischemic risk.^[46,47]^ Incorporating genotype-guided antiplatelet strategies has been shown to reduce adverse cardiovascular events in post-PCI patients, particularly among those with LOS alleles.^[47]^ Future prognostic modeling and machine-learning frameworks that integrate genotype data, platelet function testing, ischemic risk scores (e.g., DAPT, and PRECISE-DAPT), and bleeding risk tools (e.g., Academic Research Consortium-High Bleeding Risk criteria) may facilitate more precise, individualized monotherapy selection.

### 4.1. Limitations and future directions

Although the study investigated an important aspect, some limitations exist. First, the included studies were heterogeneous in terms of study design and duration of DAPT, which may have influenced the results. Second, the follow-up periods varied across studies, ranging from 1 to 5 years, which may have affected the assessment of long-term outcomes. Third, a key limitation of this meta-analysis lies in its limited ethnic generalizability. Nearly all included studies were conducted in East Asian populations (Korea, China, and Japan), which significantly restricts the broader applicability of the findings. The metabolism and clinical efficacy of clopidogrel are strongly influenced by CYP2C19 genetic polymorphisms. East Asian populations have a markedly higher prevalence of CYP2C19 LOS alleles (approximately 55–60%) compared with Caucasian (25–30%) and African (35–40%) populations.^[9,38,48]^ These alleles reduce the conversion of clopidogrel into its active metabolite and are associated with decreased platelet inhibition and higher ischemic event rates.^[47]^ Although the present meta-analysis demonstrates a consistent benefit of clopidogrel over aspirin despite this genetic profile, the concentration of evidence in East Asian cohorts limits the ability to directly extrapolate these outcomes to Western or African ancestry groups. Future clinical trials must therefore include genetically diverse populations and, where feasible, incorporate genotype-guided antiplatelet selection algorithms. Additionally, significant heterogeneity was detected for bleeding-related outcomes. This variance can be largely attributed to substantial differences in the duration of clopidogrel use (only 1 month) in the study by Watanabe et al^[18]^ prior to monotherapy initiation. Duration of DAPT affects both vascular healing dynamics and baseline bleeding susceptibility, creating potential confounding when comparing long-term monotherapy effects between aspirin and clopidogrel.^[49]^ Standardizing initial DAPT periods or stratifying analyses based on DAPT duration is recommended for future research to reduce interpretation bias. The included evidence consisted of a mixed body of study designs, combining 2 randomized controlled trials and 4 observational cohort studies. Although all cohort studies demonstrated high quality on the NOS measurement, the integration of non-randomized studies inherently introduces risks of selection bias, residual confounding, and treatment allocation influenced by clinical judgment rather than randomization. Finally, the meta-analysis was based on aggregate data rather than individual patient data, which may have limited the ability to adjust for confounding factors. The findings of this study have important implications for clinical practice. The superior efficacy of clopidogrel in reducing MACE and stroke, coupled with its comparable bleeding risk and mortality outcomes, suggests that it may be a more favorable option for long-term monotherapy in post-PCI patients. More studies should be conducted to allow an individualized choice of DAPT-Regimen, espiciallly in the presence of genetic variability in response to P2Y12-Inhibition. However, individual patient characteristics, including bleeding risk, genetic predisposition, and economic factors, should guide therapy selection. Future studies should focus on further elucidating the role of clopidogrel in diverse patient populations, particularly those with genetic polymorphisms affecting drug metabolism. Additionally, randomized trials assessing newer-generation P2Y12i, such as ticagrelor or prasugrel, as monotherapy in this context may provide further insights into optimizing antiplatelet therapy strategies post-PCI.

## 5. Conclusion

This meta-analysis provides evidence that clopidogrel monotherapy after completion of DAPT may offer superior ischemic protection and a more favorable cerebrovascular and GI safety profile compared with aspirin in patients undergoing PCI. However, these findings should be interpreted within the context of several methodological limitations, including the integration of different study designs, heterogeneity in pre-monotherapy DAPT duration, and the predominance of East Asian populations in the available evidence. Therefore, while clopidogrel appears to be a promising long-term antiplatelet strategy, individualized treatment decisions considering patient ethnicity, CYP2C19 genotype, bleeding risk, comorbidities, and cost remain essential. Further randomized trials in more ethnically diverse populations are required to support broader clinical implementation.

## Author contributions

**Data curation:** Mostafa Hossam El Din Moawad.

**Formal analysis:** Mostafa Hossam El Din Moawad.

**Supervision:** Mostafa Hossam El Din Moawad.

**Writing – original draft:** Mostafa Hossam El Din Moawad, Mahmoud Elsayed, Ibrahim Serag, Reham M. Wagih, Abdelrahman Elgharabawi, Yousr Ahmed, Abdelrahman Elkholy, Ahmed Abdelraouf, Areej M. Alsolami, Ahmed Alattar, Ibraheem M. Alkhawaldeh, Hamza A. Abdul-Hafez, Ahmed Farid Gadelmawla, Mohamed Abouzid, Mohamed O. Mohamed, Osama Bisht, Mohammed Elkholy.

**Writing – review & editing:** Mostafa Hossam El Din Moawad, Mahmoud Elsayed, Ibrahim Serag, Reham M. Wagih, Abdelrahman Elgharabawi, Yousr Ahmed, Abdelrahman Elkholy, Ahmed Abdelraouf, Areej M. Alsolami, Ahmed Alattar, Ibraheem M. Alkhawaldeh, Hamza A. Abdul-Hafez, Ahmed Farid Gadelmawla, Mohamed Abouzid, Mohamed O. Mohamed, Osama Bisht, Mohammed Elkholy.

## Supplementary Material


