# Supplementary material for: Antiplatelet dilemma: Clopidogrel or aspirin for long-term cardiovascular protection after dual antiplatelet therapy following PCI
Source: Medicine (Baltimore). 2026 Feb 28;105(9):e47773. doi: 10.1097/MD.0000000000047773 (PMC12956167; doi:10.1097/MD.0000000000047773)
Supplement: Supplementary file 1 [file medi-105-e47773-s001.docx]

| Study name | The level of representation of the affected cohort (★) | Identification of the unexposed cohort (★) | Determination of exposure (★) | Evidence that the outcome of interest was absent at the commencement of the research (★) | Comparison of cohorts based on design or assessment (max★★) | Was the follow-up duration sufficient for consequences to manifest? (★) | Evaluation of results (★) | Assessment of cohort follow-up sufficiency (★) | Quality level |
| --- | --- | --- | --- | --- | --- | --- | --- | --- | --- |
| Zhuang 2014 | ★ | ★ | ★ | ★ | ★★ | ★ | ★ | ★ | High |
| Park 2016 | ★ | ★ | ★ | ★ | ★★ | ★ | ★ | ★ | High |
| Sim 2020 | ★ | ★ | ★ | ★ | ★★ | ★ | ★ | ★ | High |
| Lan 2024 | ★ | ★ | ★ | ★ | ★★ | ★ | - | ★ | High |

Supplementary table 1: Quality assessment of cohort studies using NOS

**Supplementary Figures**


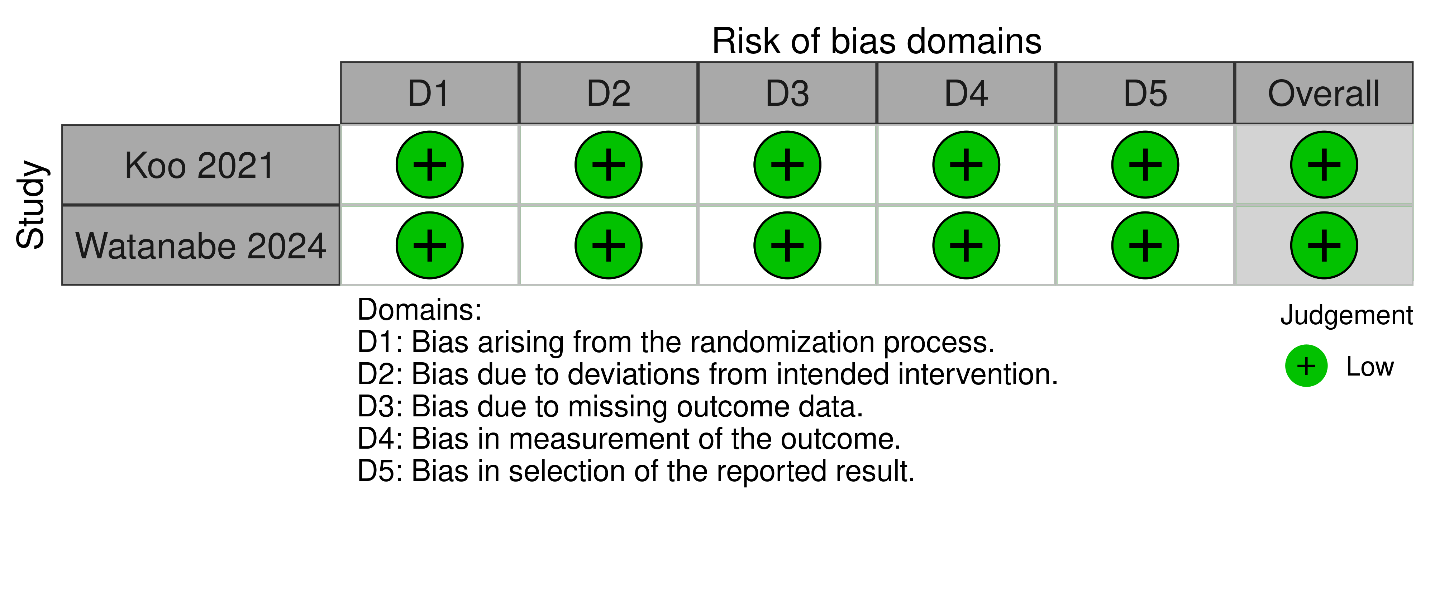


**Supplementary figure 1: Risk of bias assessment of RCTs using Rob-2**


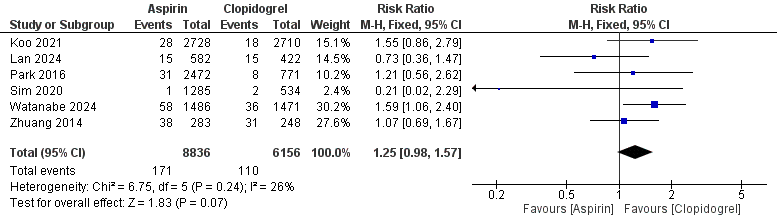


**Supplementary figure 2: Comparison between aspirin and clopidogrel monotherapy in the risk of myocardial infarction**


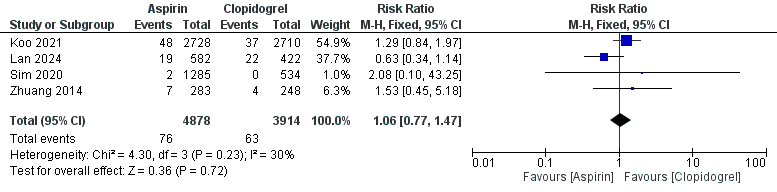


**Supplementary figure 3: Comparison between aspirin and clopidogrel monotherapy in the risk of TVR**


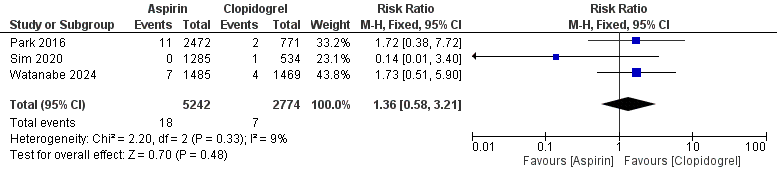


**Supplementary figure 4: Comparison between aspirin and clopidogrel monotherapy in the risk of stent thrombosis**


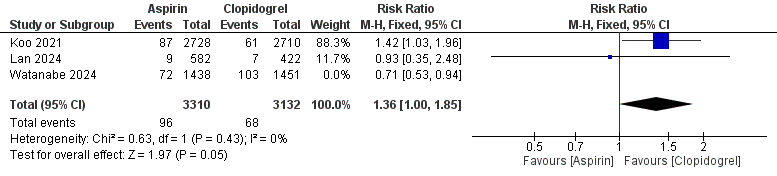


**Supplementary figure 5: Sensitivity analysis using leave-one-out of BARC bleeding 2, 3 or 5**


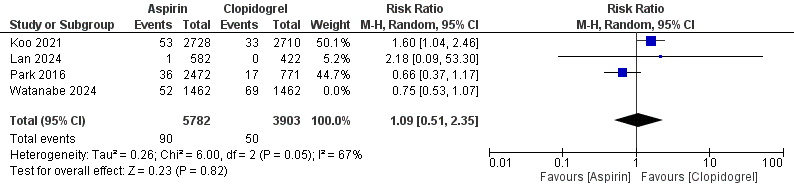


**Supplementary figure 6: Sensitivity analysis using leave-one-out of BARC bleeding 3 or 5**


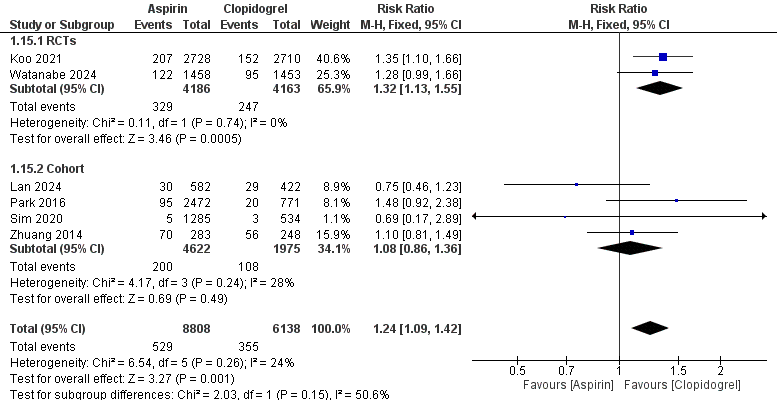


Supplementary figure 7: Comparison between aspirin and clopidogrel in the risk of MACE (sub-grouped by study design)


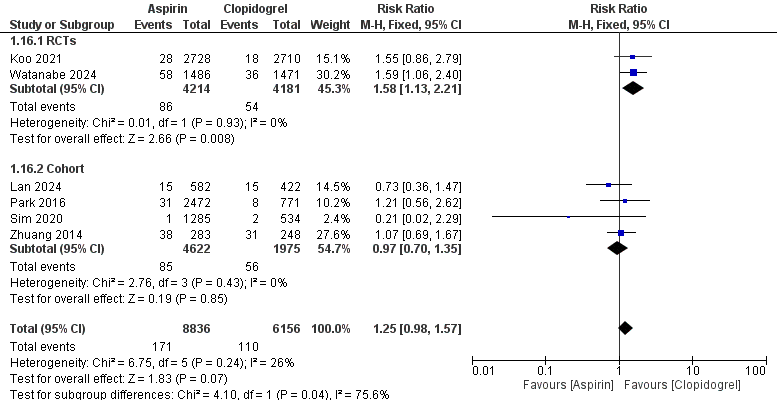


Supplementary figure 8: Comparison between aspirin and clopidogrel in the risk of myocardial infarction (sub-grouped by study design)


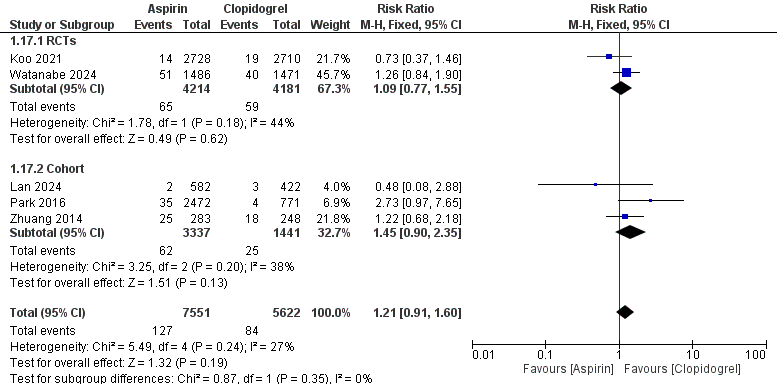


Supplementary figure 9: Comparison between aspirin and clopidogrel in the risk of cardiac mortality (sub-grouped by study design)
